# A novel oncolytic Vaccinia virus armed with IL-12 augments antitumor immune responses leading to durable regression in murine models of lung cancer

**DOI:** 10.3389/fimmu.2024.1492464

**Published:** 2025-01-07

**Authors:** Lijuan Chen, Pengju Wang, Carmela Di Gioia, Ming Yuan, Zhe Zhang, Jinxin Miao, Wenli Yan, Guanghao Zhao, Yangyang Jia, Na Wang, Zhongxian Zhang, Haoran Guo, Giulia Marelli, Louisa Chard Dunmall, Nicholas R. Lemoine, Yaohe Wang

**Affiliations:** 1 Department of Oncology, the Affiliated Cancer Hospital of Zhengzhou University & Henan Cancer Hospital, Zhengzhou, China; 2 Henan International Joint Laboratory of Lung Cancer Biology and Therapeutics, Zhengzhou, China; 3 Sino-British Research Centre for Molecular Oncology, National Centre for International Research in Cell and Gene Therapy, School of Basic Medical Sciences, Academy of Medical Sciences, Zhengzhou University, Zhengzhou, China; 4 Centre for Cancer Biomarkers and Biotherapeutics, Barts Cancer Institute, Queen Mary University of London, London, United Kingdom

**Keywords:** Vaccinia virus, lung cancer, interleukin-12, soluble PD-1, immune checkpoint inhibitor, oncolytic therapy

## Abstract

Oncolytic vaccinia viruses (VVs) are potent stimulators of the immune system and induce immune-mediated tumor clearance and long-term surveillance against tumor recurrence. As such they are ideal treatment modalities for solid tumors including lung cancer. Here, we investigated the use of VVL-m12, a next-generation, genetically modified, interleukin-12 (IL-12)-armed VV, as a new therapeutic strategy to treat murine models of lung cancer and as a mechanism of increasing lung cancer sensitivity to antibody against programmed cell death protein 1 (α-PD1) therapy. The cytotoxicity and replication of VVL-m12, VVL-h12 and control VVs were assessed in lung cancer cell lines. Subcutaneous lung cancer mouse models were established to investigate the anti-tumor activity of the viruses after intratumoral delivery in an immunocompetent disease model. Synergy with α-PD1 or a VV armed with soluble PD-1 (VV-sPD1) was investigated and functional mechanisms behind efficacy probed. Tumor-targeted VVL-m12 replicated to high levels, was cytotoxic in lung cancer cell lines. VVL-m12 demonstrated superior antitumor efficacy in subcutaneous lung cancer models compared with other VVs examined. Importantly, rational combination of VVL-m12 and PD-1 blockade worked synergistically to significantly enhance survival of animals and safely cured lung cancer with no evidence of recurrence. VVL-m12 therapy induced increased intratumoral infiltration of CD4+ and CD8+ T cells and was able to clear tumor at early time points via increased induction and infiltration of effector T cells and central memory T cells (TCM). In addition, VVL-m12 increased dendritic cell activation, induced polarization of M2 macrophages towards an M1 phenotype, and inhibited tumor angiogenesis *in vivo*. These results demonstrate that VVL-12 has strong potential as a safe and effective antitumor therapeutic for lung cancer. Importantly, VVL-12 can sensitize lung cancers to α-PD1 antibody therapy, and the combined regime creates a highly effective treatment option for patients.

## 1 Introduction

Lung cancer remains the most lethal cancer in the world, but traditional therapies are limited by intolerable side effects and poor efficacy (1). Immuno-therapeutics such as immune checkpoint inhibitors (ICIs) have emerged as a promising new approach for cancer therapy. The discovery of ICIs, including programmed death protein 1 (PD-1) and programmed death protein ligand 1 (PD-L1), has revolutionized the therapeutic landscape of lung cancer. The significant survival benefit derived from ICI treatment has established it as the mainstay first-line therapy in patients with lung cancer. However, most patients with lung cancer display primary resistance to ICI monotherapy and only 15 to 20% achieve a complete or partial response (2). Acquired resistance occurs in initially responding patients with advanced lung cancer treated with ICIs after a median progression-free survival of 4-10 months (3–6). Durable clinical responses using PD-1/L1-based therapy have been associated with a T cell-inflamed tumor microenvironment (TME) favoring the infiltration of functional cytotoxic T lymphocytes (CTLs) (7). It is now generally appreciated that driving cytotoxic immune cells into the tumor bed could significantly improve the efficacy of ICI, notably in patients displaying an immune cold phenotype (limited infiltration and/or activation of anti-tumor immune cells). This statement is supported by clinical data showing that the presence of pre-existing T cells close to the invasive tumor margin is a key determinant for achieving a long-lasting response to anti-PD-1 therapy in melanoma (8). On the basis of these studies, significant efforts to identify therapeutic approaches able to turn immunologically ‘cold’ tumors ‘hot’, by enhancing immune cell infiltration into the tumor bed, are currently being undertaken.

Oncolytic viral therapy (OVT) is proving a powerful and successful new cancer immunotherapy. OVT uses wild-type or engineered viruses designed to selectively eliminate cancer deposits and associated stromal cells directly by oncolysis, indirectly by induction of powerful immune-mediated anti-cancer responses that can promote long-term tumor immune surveillance and by targeted collapse of tumor vasculature (9, 10). The recent approval of talimogene laherparepvec (T-VEC, Imlygic), a herpes simplex virus (HSV) armed with granulocyte macrophage colony stimulating factor (GM-CSF), has paved the way in acknowledging OVT as a valid therapeutic option with a key role to play in the new era of cancer immunotherapies (11). Given the ability of OVT to induce immune reactions within the tumor, the power of OVT to sensitize tumors to ICI therapy has being postulated and the synergy between OVT and PD-1 blockade has been proven in various tumor models including colon and ovarian cancer models as well as advanced melanoma patients (12–15).

Vaccinia virus (VV) has been investigated as an OVT for treatment of many tumor types, including liver cancer, pancreatic cancer, colorectal cancer, and peritoneal carcinomatosis (16–19). VV has several inherent features that make it particularly suitable for use as an oncolytic agent for lung cancer. Vaccinia virus has an ability to replicate in a hypoxic TME, which represents the treatment-resistant fraction of many tumors, lacks requirement for a specific cell surface receptor, induces immunogenic cell death pathways that enhance immune activation within the TME, facilitates vascular collapse in the TME, has a large capacity for insertion of exogenous genes and a long-standing safety profile (17, 20–25). However, durable clinical responses to this virus remain elusive. To improve anti-tumor responses to oncolytic VV, modifications to current vectors need to be considered including a rational VV strain selection, informed gene modification to create an optimal VV to maximize the antitumor potency and safety and informed payload addition to compound the generation of potent antitumor immune activity.

We recently described a novel genetically engineered VV based on a thymidine kinase (TK)- deleted, N1L gene-deleted VV, VVLΔTKΔN1L, in which the TK and N1L gene deletions were shown to enhance not only tumor selectivity and safety, but also anti-tumor immune responses (25). A second iteration of this virus, VVLΔTK-STCΔN1L (hereafter referred to as VV CTRL) showed the additional advantage of improved viral spreading via increasing extracellular enveloped virus (EEV) production from infected cells. After viral replication in cells, a minority of the projeny virions are released as EEV (<1% of virions). EEVs are effective at mediating long-range spread of the virus due to their resistance to host cell antibodies and complement. The viral B5R protein has been shown to be important for EEV formation (26) and we have previously described that inclusion of a modified virus B5R protein, termed STC, in which the signal peptide, stalk (S), transmembrane (T) and cytoplasmic tail (C) regions (STC) of B5R are placed within the TK region can improve the ability of VV to spread within and between tumors and therefore increase *in vivo* efficacy of the virus (17). We have recently reported that arming VV CTRL with murine interleukin-21 (mIL-21) (referred to hereafter as VVL-m21) led to an improvement in the induction of antitumor immune responses in various murine models of cancer (17). Intravenously administered VVL-m21 successfully remodeled the suppressive TME, increasing the numbers of effector CD8+ T cells within the tumor, enhancing levels of circulating natural killer (NK) cells and polarizing macrophages to an M1 phenotype. Additionally, treatment with VVL-m21 sensitized tumors to the ICI α-programmed cell death protein 1 (α-PD1) (17).

Interleukin-12 (IL-12) performs a similar role to IL-21 in the immune system and is described as potentially one of the most powerful anti-tumor, pro-immune cytokines, with increased potency compared to IL-21. Oncolytic viruses (OVs) encoding IL-12 have demonstrated strong antitumor effects in preclinical models of cancers (27). IL-12 plays a pivotal role in linking adaptive and innate immune systems (28, 29). IL-12, which can induce IFN-γ production, is endogenously produced by APCs and acts upon NK and T cells and influences the differentiation of naive CD4+ T cells to a T helper 1 (Th1) phenotype (30). Thus, IL-12 serves as a master regulator of adaptive type 1 cell-mediated immunity, a critical pathway involved in the antitumor activity. Moreover, IL-12 serves as an important factor in the differentiation and survival of memory T cells (31). Additionally, IL-12 has been shown in mumerous models to exert antiangiogenic effects (32, 33). However, potentially lethal toxicity associated with systemic administration of IL-12 has precluded widespread clinical application (34–38). Intratumoral (I.T.) injection of IL-12 can circumvent the adverse effects and we propose that by using oncolytic VV to deliver IL-12 directly and specifically to the tumor site, we can mitigate the toxic side-effects associated with IL-12 and create a more powerful anti-tumor therapeutic for use in lung cancer patients. We used our next generation VV CTRL vector and armed this virus with murine or human IL-12, creating VVLΔTK-STCΔN1L-mIL12 (referred to hereafter as VVL-m12) or VVLΔTK-STCΔN1L-hIL12 (referred to hereafter as VVL-h12). Here we show that I.T. delivery of VVL-m12 can selectively control disease and durably extend survival in subcutaneous models of murine lung cancer with no toxic side effects. Additionally, it was able to effectively sensitize lung cancer to α-PD1 therapy.

## 2 Materials and methods

### 2.1 Cell lines and vaccinia viruses

CMT64 cells were established from a spontaneous alveolar lung carcinoma of a 17-month-old female C57BL/ICRF a^t^ mouse. CMT167 and 170, with increased metastatic ability, were selected and cultured from pooled lung metastases from this model (39). Mouse CMT64 cells were provided by Cell Services at Cancer Research UK, and human lung cancer cell line H460 was obtained from the American Type Culture Collection (ATCC). Lewis lung carcinoma cells (LLC, metastatic lung squamous cell carcinoma, C57BL/6) were obtained from CRUK, Clare Hall, Herts, UK. Mouse CMT167, as well as CMT170 lung cancer cell line, human lung cancer cell lines H1299 and A549 and non-cancerous cell lines NIH3T3 (murine) and NHBE (human) were obtained from the Centre for Biomarkers & Biotherapeutics, Barts Cancer Institute, Queen Mary University of London, UK. CV1 (African monkey kidney) cells were obtained from ATCC.

Construction and production of VVΔTKΔN1L, VV CTRL and VVL-m21 were described previously (17, 25). Briefly, homologues recombination was used to delete the thymidine kinase (TK) gene of Vaccinia virus Lister strain, resulting in a virus with improved tumor-specific viral replication (40). Homologous recombination was then employed to remove the N1L region, creating VVΔTKΔN1L, which further improved tumor specificity of the virus and enhanced the generation of anti-viral and anti-tumor immunity after infection (25). In VV CTRL and subsequent versions of the virus (including VVL-m21), the TK gene was replaced with a modified virus B5R protein termed STC that improves EEV production and therefore virus spread after infection (17). In these viruses the original B5R gene has been retained, but the signal peptide, stalk (S), transmembrane (T) and cytoplasmic tail (C) regions (STC) of B5R placed, as second copies, under H5 promoter control within the TK region to obtain VV CTRL. To insert cytokine payloads, murine and human IL-12 were amplified from pUNO1-mIL12 and pUNO1-hIL12 (Invivogen) using the primers mIL12_F:5'-AAGCTTATGTGTCCTCAGAAGCTAACCATCTC-3' where *HindIII* is underlined and mIL12_R:5'- ATTTAAATCCATACCACATTTGTAGAGG-3' where *SwaI* is underlined or hIL12_F:5'- AAGCTTATGTGTCACCAGCAGTTGG-3' and hIL12_R:5'- ATTTAAACCATACCACATTTGTAGAGG-3'. These fragments were inserted into the *HindIII/SwaI*-digested H5-RFP-H5 subclone prior to creation of the entire N1L shuttle vector to create N1L Left Arm-H5-H5-RFP-H5-m/hIL-12-Right Arm (17). Homologous recombination was used to insert this payload into the N1L region of the VV CTRL virus. VVΔTK-ΔN1L-msPD-1 (referred to hereafter as VV-sPD1, containing mouse soluble extracellular PD-1 fragment, msPD-1, amino acids 1-167) was constructed by the insertion of the msPD-1 into the TK region of the Lister vaccine strain of VV (VV Lister) under the control of the H5 promoter using an *in vitro* intracellular recombination technique previously described (41). The msPD-1 DNA fragment containing *SalI* and *NheI* enzyme sites was synthesized and inserted into pUC57 vector (GENEWIZ). msPD-1 DNA fragment was digested by *SalI* and *NheI*, and inserted into the TK shuttle vector containing RFP flanked by LoxP sites described previously to construct the TK-msPD-1 shuttle vector (42). The recombination of VV-ΔN1L and TK-msPD-1 shuttle vector was performed in CV1 cells. Viral purification and production were carried out as described previously (41).

### 2.2 Cell cytotoxicity assay

The cytotoxicity of the viruses in each cell line was assessed six days after infection with virus using an MTS non-radioactive cell proliferation assay kit (Promega) according to the manufacturer’s instructions. Cell viability was determined by measuring absorbance at 490 nm using a 96-well plate absorbance reader (Dynex) and a dose response curve created by non-linear regression allowing determination of an EC50 value (dose required to kill 50% of cells). A minimum of two biological repeats were carried out.

### 2.3 Vaccinia virus replication assay

Appropriate cell lines were seeded in triplicate and infected 16 hours later with virus at a multiplicity of infection (MOI) of 1 PFU/cell. Cells and supernatant (or supernatant only to assess EEV release) were collected at 24-, 48- and 72-hours post-infection and titers were determined by measuring the median tissue culture infective dose (TCID50) on indicator CV1 cells. Cytopathic effect (CPE) was determined by light microscopy seven days after infection. The Reed-Muench mathematical method was used to calculate the TCID50 value of each sample (43). Viral burst titers were converted to PFU per cell based on the number of cells present at viral infection. One-way ANOVA followed by Tukey’s multiple comparison post-test was used to assess significance. A minimum of two biological repeats were carried out.

### 2.4 Detection of IL-12 and PD-1 expression by ELISA

Appropriate cell lines were seeded in triplicate and infected 16 hours later with virus at a MOI of 1 PFU/cell. Supernatant was collected at 24-, 48- and 72-hours post-infection, IL-12 levels measured by Mouse IL-12 ELISA Kit or Human IL-12 2nd Generation ELISA Kit (eBioscience) and PD-1 levels evaluated by Mouse PD-1 DuoSet ELISA (R&D Systems).

### 2.5 *In vivo* studies

All animal studies carried out were approved by the Animal Welfare and Research Ethics Committee of Zhengzhou University (Zhengzhou, China). The efficacy study of different VVs as monotherapy in CMT64 models was carried out under the terms of the UK Home Office Project License PPL 70/6030 and subject to Queen Mary University of London ethical review, according to the guidelines for the welfare and use of animals in cancer research. Power calculations were carried out to determine required sample sizes using G*Power 3, F-test ANOVA repeated measures setting parameters of α = 0.1, power = 90%, effect size = 0.5.

### 2.6 Establishment of the *in vivo* tumor model

CMT64 (3×10^6^ cells/mouse) or LLC (1×10^6^ cells/mouse) cells suspended in 100 μl normal saline were implanted subcutaneously into the right flank of female C57BL/6 mice aged six weeks. When tumors were palpable (around 100 mm^3^), animals were stratified into treatment groups and received therapy according to the protocols. Tumor growth was measured using electronic calipers until tumors reached 1.44 cm^2^ at which point the animals were sacrificed, and tumor volume was estimated twice weekly using the formula:


$$Tumor\;Volume=\frac{\pi w^{2}l}{6}$$

Where *w* is width, *l* is length.

### 2.7 Assessment of antitumoral efficacy

Intratumoural (I.T) injections of 1×10^8^ PFU of virus or vehicle buffer in a total volume of 50 µL were introduced through a single central tumor puncture site and 3-4 needle tracts were made radially from the center; virus was injected as the needle was withdrawn. α-PD1 antibody was kindly provided by Shengdian Wang (CAS Key Laboratory of Infection and Immunity, Institute of Biophysics, Chinese Academy of Sciences, Beijing, China). α-PD1was resuspended in PBS at final concentration of 200 μg/mouse and injected via intraperitoneally (I.P) on day 1, 4 and 7 or 7, 9 and 11, as indicated in the results. Tumor growth curves were terminated on the death of the first mouse in each group, but group survival was monitored until the experimental endpoint, and Kaplan-Meier survival plots generated (one biological repeat was carried out).

#### 2.7.1 Efficacy of different VVs as monotherapy in subcutaneous lung cancer CMT64 model

When tumor volumes reached 100 mm^3^, mice were divided into four groups by matched tumor size to receive six I.T injections (in 50µl PBS) of 1×10^8^ PFU VV CTRL, VVL-m21, VVL-m12 or PBS on days 1, 3, 5, 7, 9 and 11.

#### 2.7.2 Efficacy of VVL-m12 in synergy with α-PD1 in subcutaneous lung cancer CMT64 and LLC models

When tumor volumes reached 130 mm^3^ in the CMT64 model, and 100 mm^3^ in the LLC model, mice were divided into five groups (10 mice per group), and treated according to the following protocol: α-PD1 antibody: I.P. injections of α-PD1 antibody on day 1, 4 and 7; VVL-m12/VVL-msPD1: sequential I.T injections of 1×10^8^ PFU VVL-m12 on day 1, 3, 5 and 1×10^8^ PFU of VV-msPD1 on day 7, 9, 11;VVL-m12/α-PD1 antibody: sequential I.T injections of 1×10^8^ PFU VVL-m12 on day 1, 3, 5 and I.P. injections of α-PD1 on day 7, 9, 11; VVL-m12/PBS: sequential I.T injections of 1×10^8^ PFU VVL-m12 on day 1, 3, 5 then I.P. injections of PBS on day 7, 9, 11; PBS: I.T injections of PBS on days 1, 3, 5, 7, 9 and 11.

#### 2.7.3 Re-challenge of tumor-free animals

C57BL/6 mice that underwent complete subcutaneous tumor regression following VVL-m12 (n=2) or sequential VVL-m12 and α-PD1 antibody (n=3) treatment were rechallenged with 6×10^6^ CMT64 (twice the number of cells compared to the primary tumor cell inoculation) after primary tumors had not been detected for one month. Tumor volumes were measured twice weekly.

### 2.8 Functional mechanisms for efficacy

In subcutaneous CMT64 models, when the tumor volume reached 150 mm^3^, mice were divided into three groups to receive I.T. injections of 1×10^8^ PFU VV CTRL, VVL-m12 or PBS only on day 1. On days 3, 8 and 15, subcutaneous tumors, spleens, lungs, livers, kidneys and lymph nodes were harvested from three mice in each group for further investigation, including flow cytometry (FC) analysis, IFN-γ release assay, real-time quantitative PCR, and immunohistochemistry (IHC).

### 2.9 Toxicity analysis

Blood samples were collected from the ocular venous plexus of each mouse for assessing the influence of VVs on hepatic and renal function. Serum alanine aminotransferase (ALT), aspartate aminotransferase (AST) and gamma glutamyl transpeptidase (GGT) were performed using a colorimetric method to evaluate the levels and activity of metabolic enzymes (**Elabscience**). Serum creatinine (Cr) was determined by Colorimetric Assay Kit following the instructions of the manufacturer (**Elabscience**). The level of blood urea nitrogen (BUN) was measured using a BUN detection kit (Solarbio Life Sciences) according to the protocol of the manufacturer.

### 2.10 Flow cytometry

Spleens, lymph nodes and tumors from CMT64 subcutaneous mouse models were extracted and combined with T cell culture medium (RPMI medium, 10% FBS, 1% penicillin-streptomycin, and 1% sodium pyruvate), homogenized, then pushed through a 70-μm cell strainer to create a single cell suspension. Cells were resuspended in red blood cell (RBC) lysis buffer (Sigma-Aldrich), washed in PBS, and the pellet was resuspended in T-cell medium. All fluorophore-conjugated antibodies were used at 1:200 dilution. Single-cell suspensions of spleen, tumor or lymph node were prepared in FC buffer (FB; phosphate-buffered saline (PBS) containing 1% heat-inactivated bovine calf serum (BCS)). Cells were blocked with goat serum (Beyontime) prior to incubation with fluorophore-conjugated antibody for 30 minutes. Cells were fixed in 2% formalin and analyzed using an LSRFortessa multichannel flow cytometer (Beckton Dickinson (BD) Biosciences). Raw data were analyzed using FloJo v10. Live cells were gated and from these, CD8+ T cells (CD3+CD8+), central CD8+ T (CD8+CD44^hi^CD62L^hi^, TCM) or effector memory CD8+ T (CD8+CD44^hi^CD62L^lo^, TEM) cells, CD4+ T cells (CD3+CD4+), NK cells (NK 1.1+), Treg cells (CD4+FOXP3+), dendritic cell (DC, CD11c+), and macrophages (F4/80+ CD11b+, M1: CD86+MHCII+CD206-, M2: CD206+) were identified.

### 2.11 Immunohistochemistry

CMT64 subcutaneous tumors were collected, sectioned, snap-frozen, and stored at-80°C. Frozen tissues were processed for IHC analysis of CD8+ T (BioLegend; 100702) and CD4+ T (BioLegend; 100402) cell infiltration according to the manufacturer’s instructions. Tumor tissues were fixed immediately in 10% buffered formalin phosphate and embedded in paraffin. IHC staining was performed using a labeled streptavidin-biotin method for IHC detection of CD31(Abcam; 182981).

### 2.12 Detection of microvessel density

MVD was assessed by IHC analysis using antibodies against the endothelial marker CD31 and determined according to the method of Weidner and colleagues (44). Briefly, the immunostained sections were screened at low magnifications (40× and 100×) to identify hot spots, which are the areas of highest neovascularization. Any yellow-brown stained endothelial cell or endothelial cell cluster that was clearly separate from adjacent microvessels, tumor cells, and other connective tissue elements was considered a single, countable microvessel. Within the hot spot area, the stained microvessels were counted in a single high-power (200×) field, and the average vessel count in five hot spots was considered the value of MVD. All counts were performed by three investigators in a blind manner. Microvessel counts were compared between the observers and discrepant results were reassessed. The consensus was used as the final score for analysis.

### 2.13 IFN-γ release assay

Spleens were collected from animals in the CMT64 subcutaneous mouse model and cells were separated using a 70 μm cell strainer and T cell culture medium. Red blood cells were lysed using RBC lysis buffer (Sigma-Aldrich) and cells re-suspended in complete T cell culture medium. 5×10^5^/well/100μL splenocytes were seeded into each well of a 96-well plate in triplicate. Cells were re-stimulated with 5×10^5^ mitomycin-C (MMC)-treated CMT64 or LLC cells. Separately, in order to assess immune response to the viruses, 100 μl aliquots of splenocyte suspensions were treated with Vaccinia virus B8R peptide (TSYKFESV) (GL Biochem) at a concentration of 5 mg/ml in a 96-well plate. Re-stimulated splenocytes were incubated for 72 h at 37 °C, and the supernatant was collected for quantitative detection of IFN-γ using Mouse IFN gamma uncoated ELISA kit (Invitrogen) according to the manufacturer’s instructions.

### 2.14 Statistical analysis

Statistical analysis was carried out using Graph Pad Prism 8 and SPSS 19.0 software. To compare differences between groups, one-way or two-way ANOVA with Bonferroni or Tukey’s post-test was used, and results were expressed as mean± SEM. Survival was estimated by the Kaplan-Meier method and differences between groups were analyzed using log-rank test. A value of *p* < 0.05 (*) was considered as statistically significant.

## 3 Results

### 3.1 VVL-m/h12 replicates in and is cytotoxic to murine lung cancer cells

**Figure 1:**
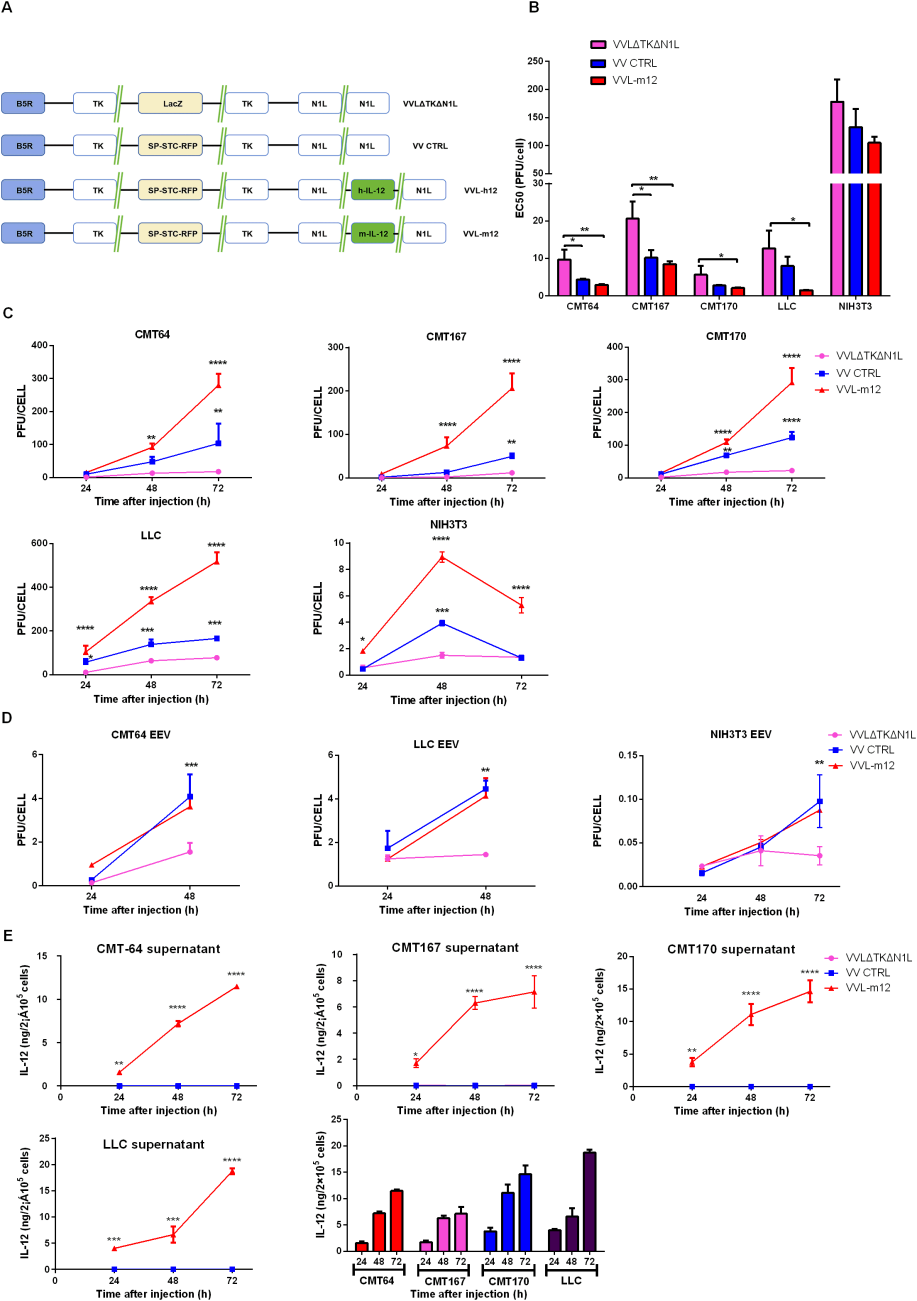
A novel tumor-selective oncolytic VVL-m12 replicates efficiently in and is cytotoxic to lung cancer cell lines. **(A)** Vaccinia virus Lister strain with deletions of the thymidine kinase (TK) and N1L genes has been described previously (25). We retained the original B5R gene and placed the signal peptide, stalk (S), transmembrane (T) and cytoplasmic tail (C) regions (STC) in the deleted four short consensus repeat (SCR) domains under an H5 promoter control within the TK region to obtain VV CTRL (17). IL-12 (murine (m) or human (h)) was incorporated into the N1L region of VV CTRL under control of the H5 promoter, creating VVL-m/h12. **(B)** Cytotoxicity of VVLΔTKΔN1L, VV CTRL and VVL-m12 against murine lung cancer LLC, CMT64, CMT167 and CMT170 cells and normal murine NIH3T3 cells. Cell death was determined by MTS assay 144 hours post-infection. EC50 values ± SEM are shown. One-way ANOVA with *post hoc* Tukey tests were used to assess significance (n=3/group). **(C, D)** Production of infectious virions after infection of murine lung cancer cell lines and normal murine cells over time. Virus production from whole cell lysates was assessed **(C)** and EEV production was determined via titration of viral supernatant from the same experiments **(D)**. Mean PFU/cell ± SEM is shown at each time-point and statistical significance determined using two-way ANOVA with Tukey’s multiple comparison post-test (n=3/group). **(E)** IL-12 expression after infection of murine lung cancer cell lines at an MOI of 1 PFU/cell. Supernatant was collected every 24 hours for 72 hours and assayed for IL-12 by ELISA. Data were normalized to cell number infected and displayed as ng/2×10^5^ cells. n=3/group. In all cases, the mean ± SEM is shown. *p<0.05; **p<0.01; ***p<0.001; ****p<0.0001.

We have previously demonstrated that VVLΔTKΔN1L has potent efficacy and tumor selectivity (25). To boost the anti-tumor potency of VV, we have previously described the generation of VV CTRL that contains a second, mutated copy of the viral protein B5R. This addition improves the production of the EEV, critical for long-range spread of the virus in the host (17). To maximize the oncolytic potential of VV, IL-m12 was incorporated into the N1L region of VV CTRL under control of the H5 promoter creating VVL-m12 (**Figure 1A**).

To determine the biological characteristics of VVL-m12 *in vitro*, cytotoxicity, replication and IL-12 expression of the virus were compared to the unarmed viruses including VVLΔTKΔN1L and VV CTRL in lung cancer cell lines. CMT64 as well as its sublines CMT167 and 170, representing late-stage invasive lung cancer, and LLC, representing metastatic lung squamous cell carcinoma, were all derived from mouse models of lung cancer (25, 39, 45, 46). A549, H460 and H1299 cells, representing the common primary malignant cell lines for human lung cancer *in vitro* studies, were also examined, but a virus expressing the human IL-12 cytokine (VVL-h12) was used in these instances.

VVL-m/h IL12 was more cytotoxic compared to VVLΔTKΔN1L in all tumor cell lines examined, as evidenced by a decrease in the infectious dose required to kill 50% of cells (EC50) (**Figure 1B**; **Supplementary Figure S1A**). VVL-m/h12 also replicated efficiently in all lung cancer cell lines examined, whereas both replication and cytotoxicity was significantly attenuated in normal cells including NIH3T3 and NHBE cell lines (**Figures 1B, C**; **Supplementary Figures S1B, D**).

Interestingly, arming VV CTRL with IL-12 led to improved replication in both murine and human lung cancer cell lines, an observation that we are as yet unable to explain. However, as arming the virus with IL-12 did not improve the cytotoxicity of the virus (compared to VV CTRL) it seems reasonable to infer that any biological effects of the IL-12 payload is mediated by its immune promoting properties as opposed to potential effects on viral replication, although this cannot be excluded as a mechanism of action.

As expected, due to the inclusion of the STC payload in VV CTRL and VVL-m/h12, both of these viruses were more competent than the parental virus VVLΔTKΔN1L, that does not contain the STC insertion, at promoting EEV release in murine and human lung cancer cell lines (**Figure 1D**; **Supplementary Figure S1E**).

To assess IL-12 expression by VVL-m/h12, lung cancer cells were infected with virus and the supernatant collected for IL-12 detection by ELISA at 24, 48 and 72 h timepoints post-infection. These results demonstrated effective production of IL-12 at all-time points in the above cell lines. The concentration of IL-12 in the supernatant reached its peak at 48 h (H1299) or 72 h post-infection. (**Figure 1E**; **Supplementary Figure S1F**).

### 3.2 VVL-m12 shows superior antitumor efficacy compared with other VVs in murine models of lung cancer

**Figure 2:**
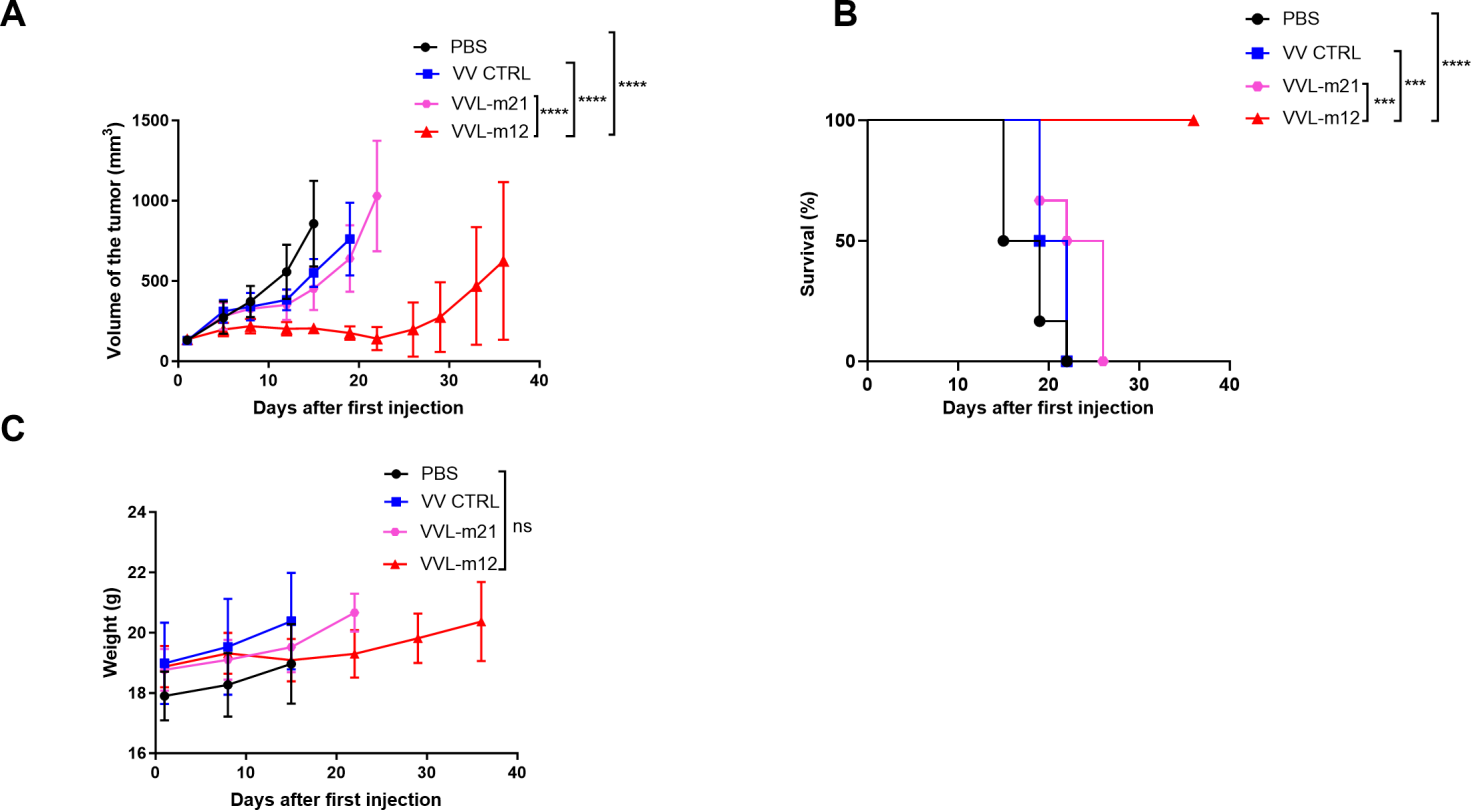
VVL-m12 is effective against CMT64 lung cancer models *in vivo*. CMT64 cells were injected into the right flank of female C57/Black 6 mice. Six-seven mice/group were injected intratumourally (I.T) with 1×10^8^ PFU VV CTRL, VVL-m21, VVL-m12, or PBS on day 1, 3, 5, 7, 9 and 11. **(A)** Tumor growth curve of CMT64 tumors treated by I.T. injection. Mean tumor size ± SEM are displayed until the death of the first mouse in each group and compared by two-way ANOVA with *post hoc* Tukey multiple comparison test. **(B)** Kaplan-Meier analysis with Log rank (Mantel-Cox) tests was used to analyse survival of the mice after treatment. **(C)** Mouse weight was assessed from the same model. Mean mouse weight ± SEM is shown, and statistical significance was determined using two-way ANOVA with *post hoc* Tukey multiple comparison test. ns: p>0.05; ***p<0.001; ****p<0.0001. PBS, phosphate-buffered saline.

CMT64 subcutaneous tumors were established in female C57/BL6 mice and the animals received intratumoral (I.T) injections of 1×10^8^ PFU of VVL-m12, VV CTRL, VVL-m21 or PBS on day 1, 3, 5, 7, 9 and 11. The dose of selected VVs was 10 times lower than the most commonly reported experimental dose of 1×10^9^ PFU/injection (47). In the CMT64 subcutaneous model, VVL-m12 was significantly more effective at controlling tumor growth and improving survival compared to VV CTRL, VVL-m21 and PBS and no toxic side effects were noted (**Figures 2A, B**). Efficacy of VVL-m12 reached its peak at day 22 after therapy after which point therapeutic response waned. There was no difference in the weight of mice between the VVL-m12 and other groups (**Figure 2C**), suggesting that IL-12 did not add toxicity to the therapeutic regime.

### 3.3 VVLm-12 sensitizes lung cancer to checkpoint inhibitor α-PD1 antibody and VV-msPD1

Monoclonal antibodies that block the inhibitory PD-1 or PD-L1 pathways have shown remarkable efficacy in patients suffering with lung cancer. As an alternative strategy for antitumor therapy, a soluble form of PD-1 containing the extracellular domain of the membrane bound molecule has shown promising potential in animal models and clinical antitumor immunotherapy (48, 49). VV-msPD1 was rationally engineered by inserting a soluble PD-1 peptide in the TK region (**Supplementary Figure S2A**). VV-msPD1 replicated efficiently in and was selectively cytotoxic to all lung cancer murine cell lines examined (**Supplementary Figures S2B, D, E**). Effective production of PD-1 in the supernatant of lung cancer cells after infection of VV-msPD1 was detected by ELISA at 24, 48 and 72 h timepoints post-infection (**Supplementary Figures S2C, F**).

**Figure 3:**
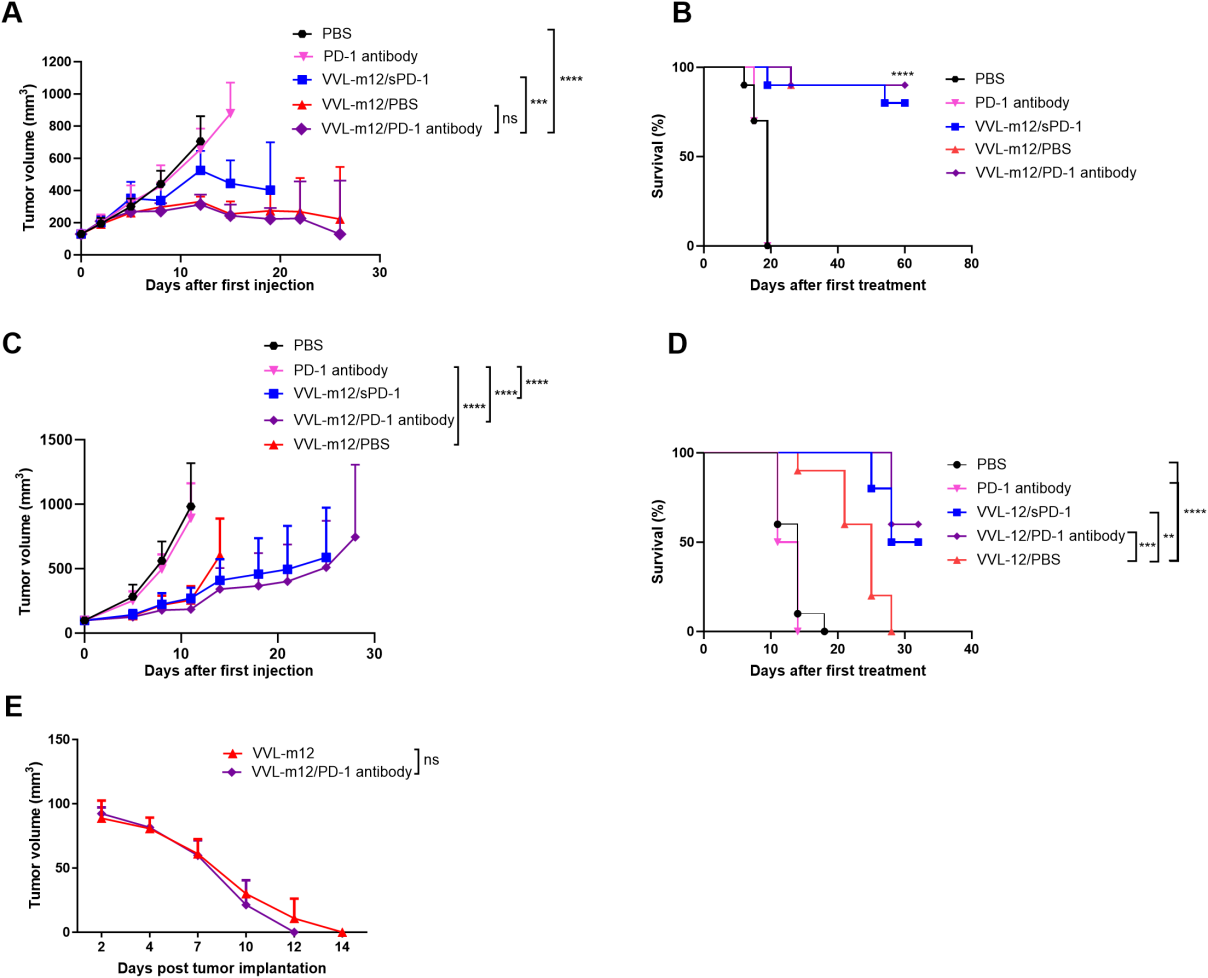
VVL-m12 sensitizes lung cancer to checkpoint inhibitor α-Programmed cell death protein 1 (α-PD1) and VVΔTK-ΔN1L-msPD-1 (VV-sPD1) resulting in long-term protection against disease recurrence. CMT64 **(A, B)** and LLC **(C, D)** subcutaneous tumors were established and treated according to the following protocol: PBS: 100ul PBS injected intratumourally (I.T) on days 1, 3, 5, 7, 9 and 11; PD-1 antibody:α-PD1 antibody delivered intraperitoneally (I.P) on days 1, 4 and 7; VVL-m12/sPD1: Sequential intratumoral injection. (I.T.) of VVL-m12 on day 1, 3, 5 and VV-msPD1 on day 7, 9, 11; VVL-m12/PD-1 antibody: Sequential I.T. injections of VVL-m12 on days 1, 3, 5 and I.P. injections of α-PD1 antibody on day 7, 9, 11; VVL-m12/PBS: Sequential I.T. injections of VVL-12 on day 1, 3, 5 and I.P injections of PBS on day 7, 9, 11 (n=9-11/group). **(A, C)** Tumor sizes were measured twice weekly and the mean ± SEM is shown. Significance was assessed using two-way analysis of variance with Tukey’s multiple comparison post-test. **(B, D)** Kaplan-Meier survival analysis with log-rank tests (Mantel-Cox) tests were used to assess survival. For **(B)**, the statistical advantage of the three VV-m12 groups compared to treatment with α-PD1 antibody or PBS is shown by ****. **(E)** CMT64 subcutaneous mice that underwent complete subcutaneous tumor regression following VVL-m12 (n=2) monotherapy or sequential VVL-m12 and α-PD1 antibody (n=3) treatment were rechallenged with 6×10^6^ CMT64 (twice the number of cells compared to the primary tumor cell inoculation) after primary tumors had not been detected for 1 month. Tumor volumes were measured twice weekly, and significance was determined using two-way ANOVA with Tukey’s multiple comparison post-test. ns: p>0.05; **p<0.01; ***p<0.001; ****p<0.0001; PBS, phosphate-buffered saline.

We investigated whether the combination of VVL-m12 with α-PD1 antibody or the sequential provision of VVL expressing IL-m12 then VV-msPD1 was able to enhance the antitumor efficacy in murine lung cancer. Both CMT64 and LLC lung cancer models were established and treated according to the protocols described in the methods. The efficacy of α-PD1 antibody treatment alone was limited with no significant difference compared with PBS in CMT64 animals (**Figures 3A, B**). Using mIL-12 armed viruses in the treatment regime showed a significant advantage in this model, and the addition of PD1 antibody may be beneficial. 9/10 animals treated with VV-m12/VV PD1 antibody cleared tumor completely by day 30, while 8/10 animals treated with VV-mIL12/PBS or VV-m12/VV-msPD1 cleared tumors by day 50. These animals were kept for their natural lifespan (around 1 year further) and no tumor recurrence was noted. Superiority of IL-12 was also noted using an aggressive LLC model compared with both PBS and α-PD1 antibody treatment. Overall survival was significantly improved after treatment with these viruses and targeting PD1 expression using either VV-msPD1 or α-PD1 antibody in combination with virus was significantly more effective than treatment with VV-mIL12 alone (**Figures 3C, D**). Overall, this data suggests that arming the virus with IL-12 and including a further therapeutic boost with either α-PD1 or a VV expressing soluble PD1 (VV-msPD1) creates a highly effective therapeutic regime for long-term elimination of lung cancer in murine models.

### 3.4 VVL-m12 treatment results in long-term protection against disease recurrence

Successful immunotherapeutic regimes aim not only to inhibit the primary tumor, but also to induce durable long-term immunity to prevent tumor recurrence. Thus, mice that had effectively cleared primary tumors in sequential VVL-m12 and α-PD1 antibody as well as VVL-m12 monotherapy groups were re-challenged with CMT64 cells 30 days after the primary tumors had completely cleared. Treatment with both therapeutic regimes resulted in long-term antitumor efficacy to CMT64 lung cancer cells as evidenced by rapid tumor clearance that necessitated no further immunotherapy. Although sequential VVL-m12 and α-PD1 antibody treatments were able to clear the secondary tumor more quickly, the difference between the two groups was not significant (**Figure 3E**).

### 3.5 VVL-m12 treatment efficacy relies primarily on CD8+ TIL and CD4+ TIL

**Figure 4:**
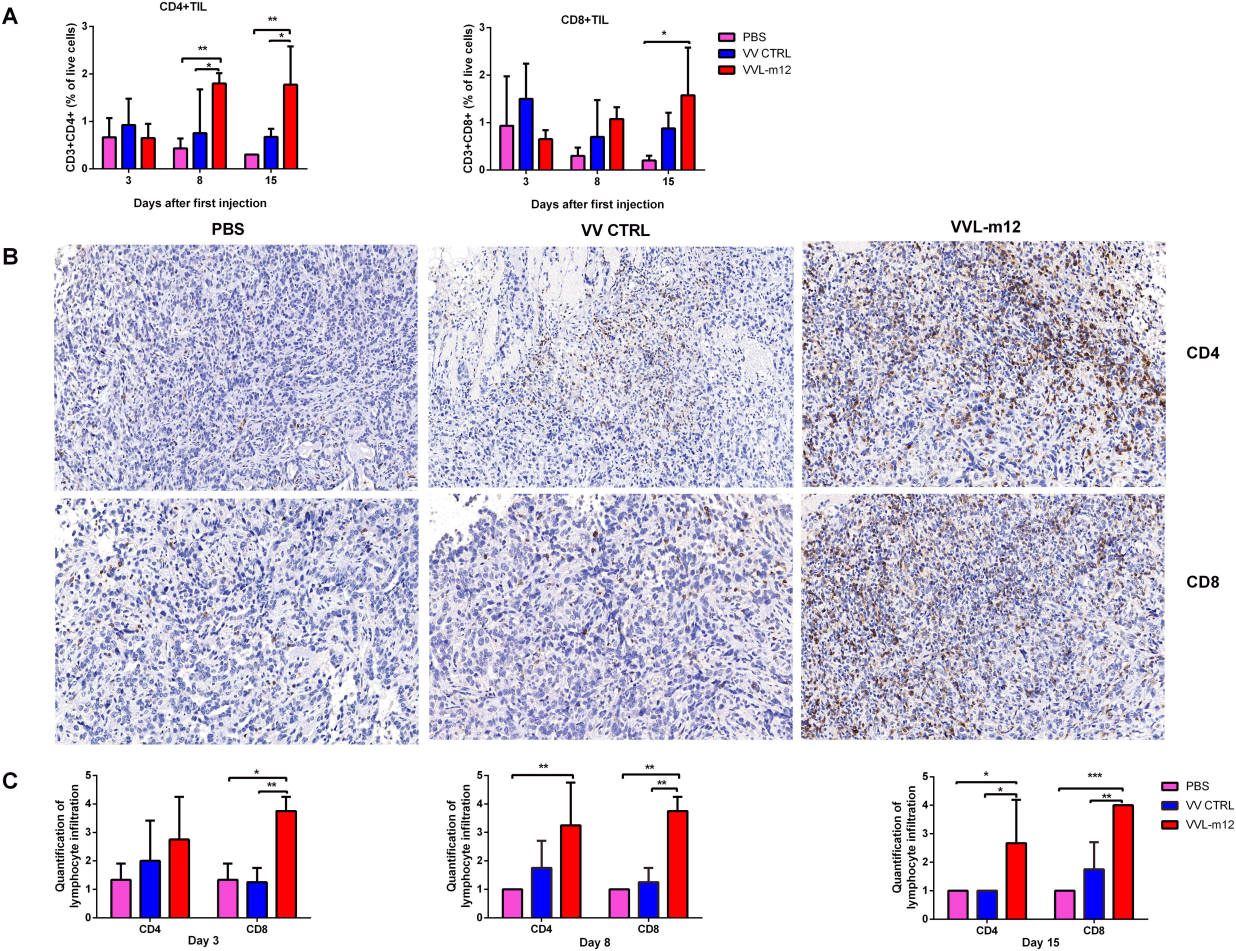
VVL-m12 therapy induces infiltration of CD4+ T and CD8+ T cells into the CMT64 tumor. Subcutaneous CMT64 mice were treated with one I.T. injection (1×10^8^ PFU) of VV CTRL, VVL-m12 or PBS on day 1. Tumors were harvested 3, 8 and 15 days after injection and analyzed using flow cytometry (FC) and immunohistochemical staining (IHC) (n= 3/group). **(A)** CD4+ and CD8+ populations as a percentage of live cells in tumor tissue of treated mice assessed by gating on CD3+CD4+ and CD3+CD8+, respectively. **(B)** Representative images of IHC staining for CD4+ T and CD8+ T cells in CMT64 subcutaneous tumors collected on day 8 (×200), n=3/time point. **(C)** CD4+ T and CD8+ T cells were counted in five high-power fields (HPFs) from each tumor section (×200). Quantitative scores of lymphocyte infiltration within the tumor were assessed on days 3, 8 and 15 after therapy. The extent of positive cells was categorized into the following four grades: 1, <15 cells/HPF; 2, 16-30 cells/HPF; 3, 31-45 cells/HPF; 4, > 45 cells/HPF. Significance was determined by two-way ANOVA with Tukey’s multiple comparison test. *p<0.05; **p<0.01; ***p<0.001. PBS, phosphate-buffered saline.

The infiltration of immune cells into tumors, referred to as tumor-infiltrating lymphocytes (TILs), consequent to immunotherapy is a strong predictor of treatment success. To investigate TILs in response to our treatment regime, a CMT64 lung cancer subcutaneous model was established and treated once I.T. with PBS or VVs (1×10^8^ PFU). Tumor tissue was collected on days 3, 8 and 15 for analysis using flow cytometry (FC) and immunohistochemistry (IHC). At all time-points, treatment with VVL-m12, but not VV CTRL or PBS resulted in significant increase in CD4+ TIL, and more predominantly CD8+ TIL, in the tumor tissue demonstrating that VVL-m12 acts primarily via adaptive CD4+ TIL and CD8+ TIL to mediate antitumor effects in this model (**Figures 4A–C**).

### 3.6 VVL-m12 treatment promotes memory T cell formation to achieves durable tumor clearance

**Figure 5:**
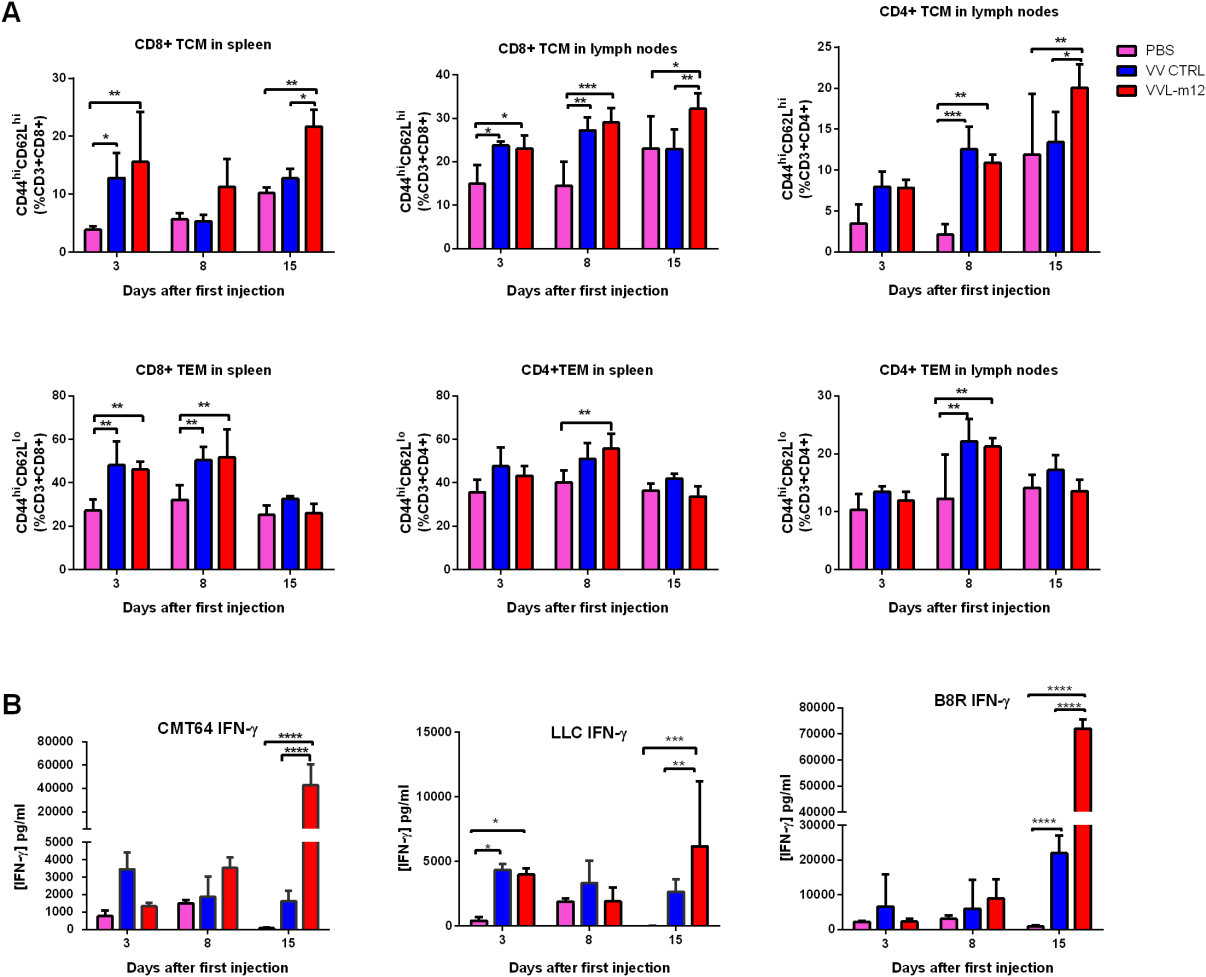
VVL-m12 promotes memory T cell formation and induces robust and durable antitumor adaptive immune responses. CMT64 tumors were established as previously and treated once with 1×10^8^ PFU VV CTRL or VVL-m12 or PBS. **(A)** CD8+TCM, CD8+TEM, CD4+TCM and CD4+TEM populations as percentage of respective populations in splenocytes or lymph nodes of treated mice were determined using flow cytometry (FC) (n=9/group). **(B)** After treatment, the response of splenocytes to mitomycin-C killed tumor cells or viral protein B8R epitope was examined *ex vivo* using tumor cells restimulation assays on days 3, 8 and 15 after the first treatment. Interferon (IFN)-γ production in response to stimulation was determined after incubation for 72 hours using ELISA. Mean production ± SEM is shown and a two-way analysis of variance (ANOVA) with Tukey’s multiple comparison post-test used to determine statistical significance. *p<0.05; **p<0.01; ***p<0.001; ****p<0.0001.

TCM subsets including CD8+TCM (CD44^hi^CD62L^hi^ CD8+) and CD4+TCM (CD44^hi^CD62L^hi^CD4+), and TEM cells consisting of CD8+TEM (CD44^hi^CD62L^lo^CD8+ cells) and CD4+TEM (CD44^hi^CD62L^lo^CD4+ cells) were analyzed using FC on day 3, 8 and 15 after treatment according to gating criteria for these cells (**Supplementary Figures S4A, B**). VVL-m12 significantly promoted CD8+ and CD4+ TCM formation by day 15 compared to VV CTRL or PBS (**Figure 5A**). CD8+ TEM and CD4+ TEM populations, which are critical for the early phase antitumor immune responses, increased on days 3 and 8 post-treatment. VVL-m12 treatment was able to clear tumor more quickly via induction of TEM together with TCM and more consistently owing to a greater TCM influx compared with VV CTRL (**Figure 5A**).

We examined interferon (IFN)-γ production from splenocytes isolated from these models and found that VVL-12 treatment was able to modestly induce IFN-γ production from *ex vivo* re-stimulated splenocytes at 3 or 8 days after VV therapy, but significantly enhanced *ex vivo* IFN-γ production by splenocytes stimulated with mitomycin C-treated CMT64 and LLC lung cancer cells harvested at 15 days post-treatment. The same trend of IFN-γ release occurred after restimulation with the viral immunogen B8R (**Figure 5B**).

### 3.7 Effect of VVL-m12 therapy on innate and other immune compartments

**Figure 6:**
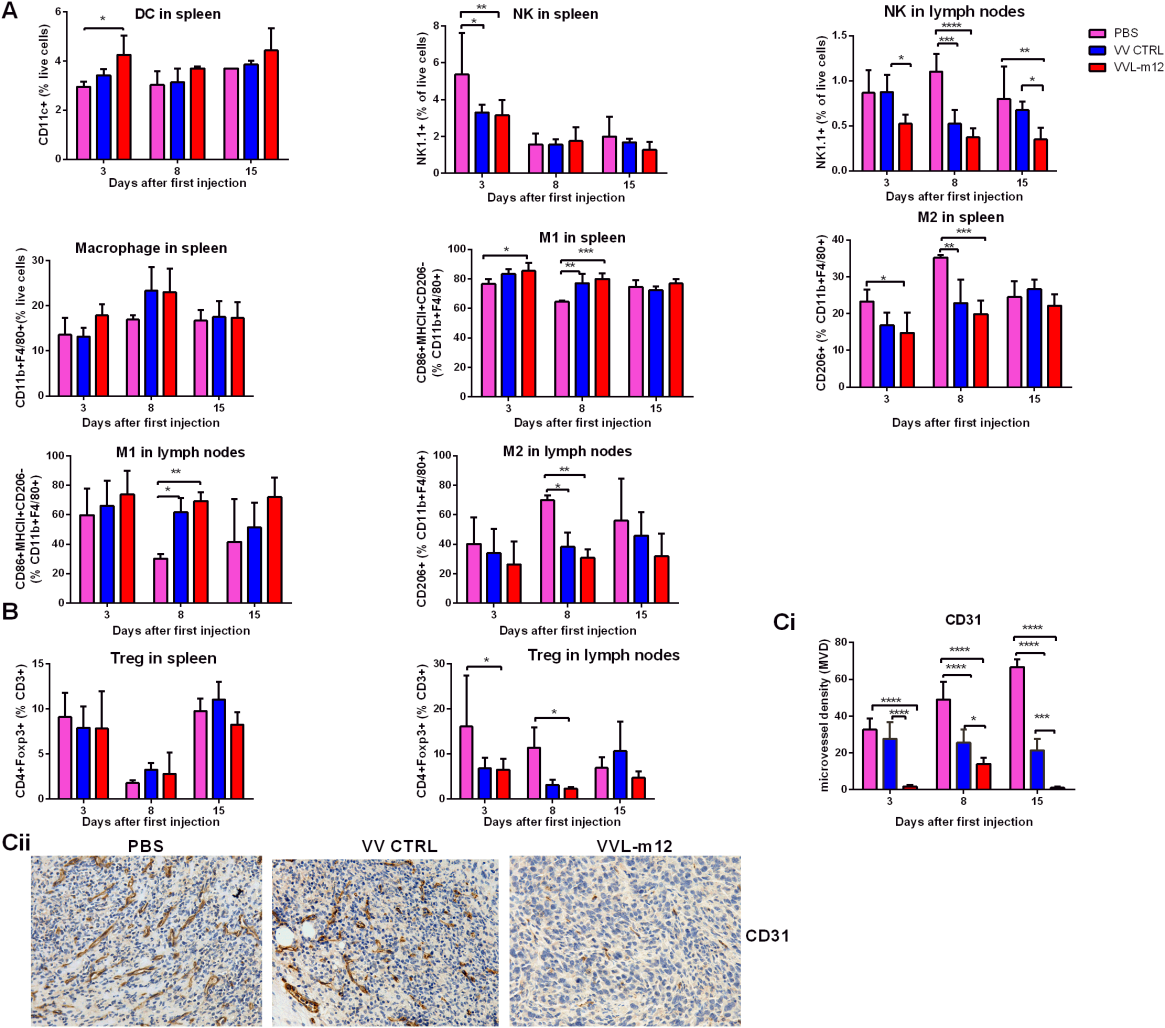
VVL-m12 induces early innate immunity, Treg down-regulation and antiangiogenic effects against murine lung cancer *in vivo*. CMT64 tumors were established as previously and treated once with 1×10^8^ PFU VV CTRL or VVL-m12 or PBS. Spleen and lymph nodes were harvested 3, 8 and 15 days after treatment. **(A)** VVL-m12 treatment results in a significant increase of splenic dendritic cells (DC) and M2 to M1 macrophage polarization. Natural killer (NK) cell numbers in the spleen were decreased after VV-m12 treatment. Using FC, tissue infiltrated DCs, NK cells and macrophages are defined as CD11c+, NK1.1+ and CD11b+F4/80+ cells, respectively. M1 macrophages are CD86+MHCII+CD206- CD11b+F4/80+ cells, whereas M2 macrophages are CD206+CD11b+F4/80+ cells. Percentage of innate cells was shown, and significance was determined using a two-way ANOVA with Bonferroni post-test (n=3/group). **(B)** VVL-m12 significantly reduced Treg cells (CD4+FOXP3+) in lymph nodes but did not alter Treg cells in spleen. Percentage of cell populations was shown, and significance was analysed using a two-way ANOVA with Bonferroni post-test (n=3/group). *p<0.05; **p<0.01; ***p<0.001; ****p<0.0001. **(C)** Immunohistochemical analysis of vascularity in CMT64 tumors after treatment. Microvessel density (MVD) was assessed using IHC and the average vessel count in 5 areas was counted manually. The immunostained sections were initially screened at low magnifications (40× and 100×) to identify hot spots, and within these areas, the stained microvessels were counted in a single high-power (200×) field. The mean value of MVD ± SEM was compared, and the significance was evaluated via two-way ANOVA with Bonferroni post-test (n=3/group). (Ci) The average vessel count in five hot spots was considered the value of MVD, and mean value of MVD ± SEM was shown on days 3, 8 and 15 after the first treatment. (Cii) Reprentative images of tumor vascularity were shown at day 15 from PBS, VV CTRL and VVL-m12. *p<0.05; **p<0.01; ***p<0.001; ****p<0.0001.

Using the same model described above, innate immune cells in the spleen and lymph nodes were examined at 3, 8 and 15 days post-treatment. VVL-m12 treatment did not enhance NK cell counts in the spleen (**Figure 6A**). We demonstrated that VVL-m12 treatment significantly increased DC populations on day 3, reduced Treg cells from day 3 to 8 (**Figure 6B**), and was effective at re-polarization of M2 macrophages to an M1 phenotype on day 3 and 8 *in vivo* and *in vitro* (**Figure 6A**; **Supplementary Figures S3A, B**, **S4A**), all of which may contribute to a more powerful antitumor potential in the early stage of antitumor immunity.

### 3.8 VVL-m12 inhibits tumor angiogenesis

Both VV and IL-12 have been reported to have powerful anti-angiogenic effects that may provide a further anti-tumor mechanism to the treatment. To assess this, MVD was determined from CMT64 tumor sections treated once with virus or PBS. Seciotns were stained with an antibody reactive to CD31 and the number of the micro-vessels per high-power field (HPF) counted. Three days after therapy, MVD in PBS, VV CTRL, and VVL-m12 groups was 32.67± 3.53, 27.67± 5.24 and 1.75± 0.48, respectively. The difference between the three groups was more significant over time using one-way ANOVA analysis (MVD in PBS, VV CTRL, and VVL-12 at day 8: 49± 5.57, 25.5± 3.66 and 14± 2; day 15:66.67 ± 2.40, 21.5 ± 3.01 and 1 ± 0.41). Quantification revealed VVL-12 treatment resulted in significant decrease in vascularity compared with PBS and VV CTRL (**Figure 6C**).

### 3.9 I.T delivery of VVL-m12 is non-toxic and tumor specific

As toxicity reactions have been associated with systemic delivery of IL-12, we evaluated the safety associated with I.T (localized) administration of VVL-m12 compared with VV CTRL and PBS. Liver and renal function was examined after VVL-12 (1×10^8^ PFU), VV CTRL (1×10^8^ PFU) or PBS were administered once into subcutaneous CMT64 tumors. Liver toxicity was assessed by measuring ALT, AST and GGT. Renal toxicity was evaluated by measuring Cr and BUN. No elevations were noted in any enzymes indicating that liver and renal toxicity was not a side-effect associated with VVL-m12 treatment (**Supplementary Figures S5A, B**). Furthermore, VVL-m12 also exhibited strong tumor selectivity *in vivo*. Tumor tissues, liver, lung, and kidney were examined after I.T. administration of VVL-m12 into C57/BL6 mice bearing subcutaneous CMT64 lung cancer on days 3, 8 and 15. Vaccinia virus late transcription factor 2 (VLTF-2) gene, detected after VVL-m12 treatment, was confined to lung cancer tissue and absent from liver, kidney and normal lung tissues (**Supplementary Figure S5C**).

## 4 Discussion and conclusion

Tumor-targeted OVs, widely acknowledged as a validated immunotherapeutic strategy, specifically infect and lyse tumor cells, release tumor-associated antigens (TAAs) and induce antitumor immune responses. VVs are powerful stimulants of the innate and adaptive immune system and during clinical studies no limiting toxic side effects have been noted, making them ideal agents for treatment of cancer (16, 50). To date, the use of VVs alone has proved unsuccessful in numerous preclinical and clinical studies and this is likely due to sub-optimal immune activation and their premature clearance preventing their oncolytic effects (16, 17, 50). As such, treatment strategies should be optimized to address these limitations, with emphasis placed on improving VV design or rational combination strategies to overcome the strongly immunosuppressive TME. We have recently described novel oncolytic VVs, based on the Lister strain backbone that we found to have the most acceptable therapeutic index compared with other strains of VV, including the commonly used WR strain, originally reported as the most potent strain of VV. Deletion of the TK and N1L genes significantly enhanced both innate and adaptive immune responses systemically and within the TME and EEV production of virions subsequent to cell infection was further improved by additional viral gene modifications to ensure effective spreading of the virus within and between tumor deposits (17, 25).

IL-12 is a cytokine with potent antitumor activity, but its systemic application is limited by toxic inflammatory responses. Nevertheless, IL-12’s pleiotropic activity, *i.e.*, its ability to engage multiple effector mechanisms and reverse tumor-induced immunosuppression, continues to entice cancer researchers (51–53). The development of strategies which maximize IL-12 delivery to the TME while minimizing systemic exposure are of increasing interest. Localized delivery systems are supporting an IL-12 renaissance which may finally allow this potent cytokine to fulfill its considerable clinical potential (54). Unfortunately, localized IL-12 delivery is unable to generate significant systemic antitumor immunity for “systemic” cancers and strategies for re-designing IL-12 seem critical for a robust antitumor response. Diverse delivery strategies for IL-12-based cancer immunotherapies, including immuno-cytokine fusions, polymeric nanoparticles, nucleic acid-based delivery and IL-12-transduced cells such as chimeric antigen receptor (CAR) T cells have demonstrated antitumor immunity with reduced adverse events in preclinical studies, however, significant, durable responses have been elusive (55–61). OVs are promising vectors for IL-12 due to their selective killing of tumor cells and ability to stimulate the host immune system, and have been tested in numerous early phase clinical studies, but the antitumor activity thus far has been limited. In clinical trials, injection of Ad-RTS-hIL-12 to two peritumoral sites immediately after resection showed limited antitumor activity against human glioma with severe adverse events including cytokine release syndrome (62). I.T injection of HF10, which was derived from a herpes simplex virus-1, in combination with gemcitabine and erlotinib demonstrated survival benefit for patients with locally advanced pancreatic cancer, however, severe adverse events including Grade III myelosuppression, perforation of the duodenum, and hepatic dysfunction limited clinical application (63).

Despite advances in prevention measures and treatment options for lung cancer, it remains the leading cause of cancer death in the world and novel treatment regimens are urgently sought. While conventional ICIs, such as anti-PD1, are commonly used for the treatment of lung cancer, efficacy is not universal and novel immunotherapeutic or combination therapies that augment T cell responses are urgently needed (64). Pelareorep, which was a proprietary isolate of the reovirus Type 3 Dearing strain, did not show survival benefit in patients with lung cancer (65). The combination regimen using vaccine TG4010 (MVA-MUC1-IL2) and first-line chemotherapy in advanced-stage non-small cell lung cancer was found safe and effective in improving progression-free survival (TG4010: 5.9 months vs Placebo: 5.1 months, p = 0.019) (66). Yet, only a limited percentage of patients achieve objective clinical responses through these novel treatments, leaving significant room for improvement of lung cancer immunotherapy (64–66).

VV has some potential advantages over other viral vectors. VV is a DNA virus with an extensive safety profiles in humans as this virus has been used in millions of people for smallpox eradication across the world (67). Compared to adenoviral vectors, the virion particle size and DNA organization of VV allows insertion of multiple transgenes with less deleterious effects on subsequent viral DNA replication, virion packaging and dissemination (68). In contrast to other viral vectors which replicate in the nucleus and depend on host transcription factors for DNA replication, the life cycle of VV is entirely located in the cytoplasm of infected host cells (69). In addition, the infectious virion is packaged with pre-transcribed early ATP and viral gene mRNA so consequently viral replication is initiated early after infection and the life cycle of VV is shorter than other OVs (70). Here, we report the arming of our next generation VV with mIL-12 to create a powerful therapeutic molecule suitable for I.T injection into lung cancer.

The lung cancer subcutaneous tumor models we developed were based on the use of CMT64 and LLC cell lines, which were originally derived from murine models of spontaneous lung cancer, and therefore resemble human lung cancer in many respects (25, 39). Here, we report the biological characteristics and anti-tumor efficacy of VVL-m12 against lung cancer. *In vitro*, all of the cell lines examined supported viral replication and the virus was cytotoxic to the cells. Interestingly, IL-12 armed viruses appeared to replicate more effectively compared to unarmed viruses (VV CTRL). IL-12 has not previously been shown to have a direct effect on viral replication *in vitro*. However, an improved cytotoxic effect of VV-m/h12 was not noted in comparison to VV CTRL, suggesting that *in vivo*, the direct immune-promoting effects of IL-12 are likely to be responsible for the improved anti-tumor immune effect rather than the enhanced replicative ability of the virus.

In clinical trials, tumor-infiltrating T cells and PD-L1 expression are both known to indicate the potential for response to immunotherapy (66, 71, 72). By using an oncolytic VV that is designed to attract T cells with high efficiency and upregulate PD-L1 as a consequence of IFN-γ induction after VV infection, the transformation of anti-PD1-resistant tumors into sensitive tumors is feasible (17, 25). As an alternative perspective, VV is most effective as an immune therapy, yet it induces immunosuppressive factors for its own protection. As a result, the combination of ICIs with VVs should enhance its efficacy. The combination of oncolytic VVs and ICI therapy is currently being explored for a number of cancers (17, 73) and indeed we demonstrated that rational combination of VVL-m12 and PD-1 blockade worked synergistically to enhance therapeutic efficacy in lung cancer.

Treatment with a combination of VVL-m12 and α-PD1 antibody or VVL-m12 alone also resulted in rejection of tumors after re-challenge, confirming the development of effective long-term immunity against TAAs and long-term survival benefit of animals.

Both VVL-m12 and VV CTRL were able to induce CD4+ and CD8+ T cell infiltration into the tumor, but VVL-m12 was able to significantly enhance the production of antitumor effector CD8+T cells and CD4+T cells within the tumor and to effectively stimulate IFN-γ production, suggesting that the potential of VVL-m12 at inducing adaptive immune responses systemically was superior to VV CTRL.

Memory T cells which play a pivotal role in defending against pathogens are generally subdivided into three populations: effector memory T cells (TEM), central memory T cells (TCM) and tissue-resident memory T cells. Human memory T cells share many phenotypic characteristics with their murine counterparts. TEM rapidly exhibits effector functions. CD4+TCM facilitate immune surveillance of antigens collected via lymphatic drainage or DCs from peripheral tissues, rapidly generating a robust and long-lived effector T cell pool upon secondary encounter of cognate antigens (74). The generation and maintenance of CD8+TCM is more crucial to long term host survival than CD4+TCM (75). Here, we found that VVL-m12 treatment was able to induce TEM for rapid effector function and promoted CD8+TCM formation and sustained IFN-γ production to achieve durable tumor clearance.

Innate immune cells, such as macrophages, DCs and NK cells, are crucial for early defense in immune responses. Macrophages are a heterogeneous population of phagocytic cells with distinct functional properties and can be considered to have tumor-promoting M2-like or antitumor M1 phenotypes (17). We demonstrated that VVL-m12 treatment significantly enhanced splenic DC populations and favored the repolarization of M2 macrophages to an M1 phenotype. In addition, reduced intratumoral Treg cell burdens after treatment. Both VV and IL-12 have previously been shown as anti-angiogenic agents (24, 32, 33, 76, 77) and here we showed that their combination indeed had a significant anti-angiogenic effect on tumors.

Together, these findings demonstrate that a rationally constructed Vaccinia virus armed with IL-m12 shows great promise as a novel therapeutic for lung cancer and is able to enhance long-term tumor clearance through modulation of adaptive and innate immune responses and anti-angiogenesis. This VV may expand the therapeutic landscape for both lung cancer and ICI treatments.

## Data Availability

The raw data supporting the conclusions of this article will be made available by the authors, without undue reservation.
