# Correction: A novel oncolytic Vaccinia virus armed with IL-12 augments antitumor immune responses leading to durable regression in murine models of lung cancer

**DOI:** 10.3389/fimmu.2026.1812288

**Published:** 2026-03-04

**Authors:** Lijuan Chen, Pengju Wang, Carmela Di Gioia, Ming Yuan, Zhe Zhang, Jinxin Miao, Wenli Yan, Guanghao Zhao, Yangyang Jia, Na Wang, Zhongxian Zhang, Haoran Guo, Giulia Marelli, Louisa Chard Dunmall, Nicholas R. Lemoine, Yaohe Wang

**Affiliations:** 1 Department of Oncology, the Affiliated Cancer Hospital of Zhengzhou University & Henan Cancer Hospital, Zhengzhou, China; 2 Henan International Joint Laboratory of Lung Cancer Biology and Therapeutics, Zhengzhou, China; 3 Sino-British Research Centre for Molecular Oncology, National Centre for International Research in Cell and Gene Therapy, School of Basic Medical Sciences, Academy of Medical Sciences, Zhengzhou University, Zhengzhou, China; 4 Centre for Cancer Biomarkers and Biotherapeutics, Barts Cancer Institute, Queen Mary University of London, London, United Kingdom

**Keywords:** Vaccinia virus, lung cancer, interleukin-12, soluble PD-1, immune checkpoint inhibitor, oncolytic therapy

There was a mistake in **Figure 4** as published. During the figure assembly process, a representative image from the VV CTRL group was accidentally inserted into the PBS group of **Figure 4B** (CD4+ Immunohistochemistry). This image has now been replaced with a correct and representative image from the PBS group. The corrected **Figure 4** appears below.

**Figure 4:**
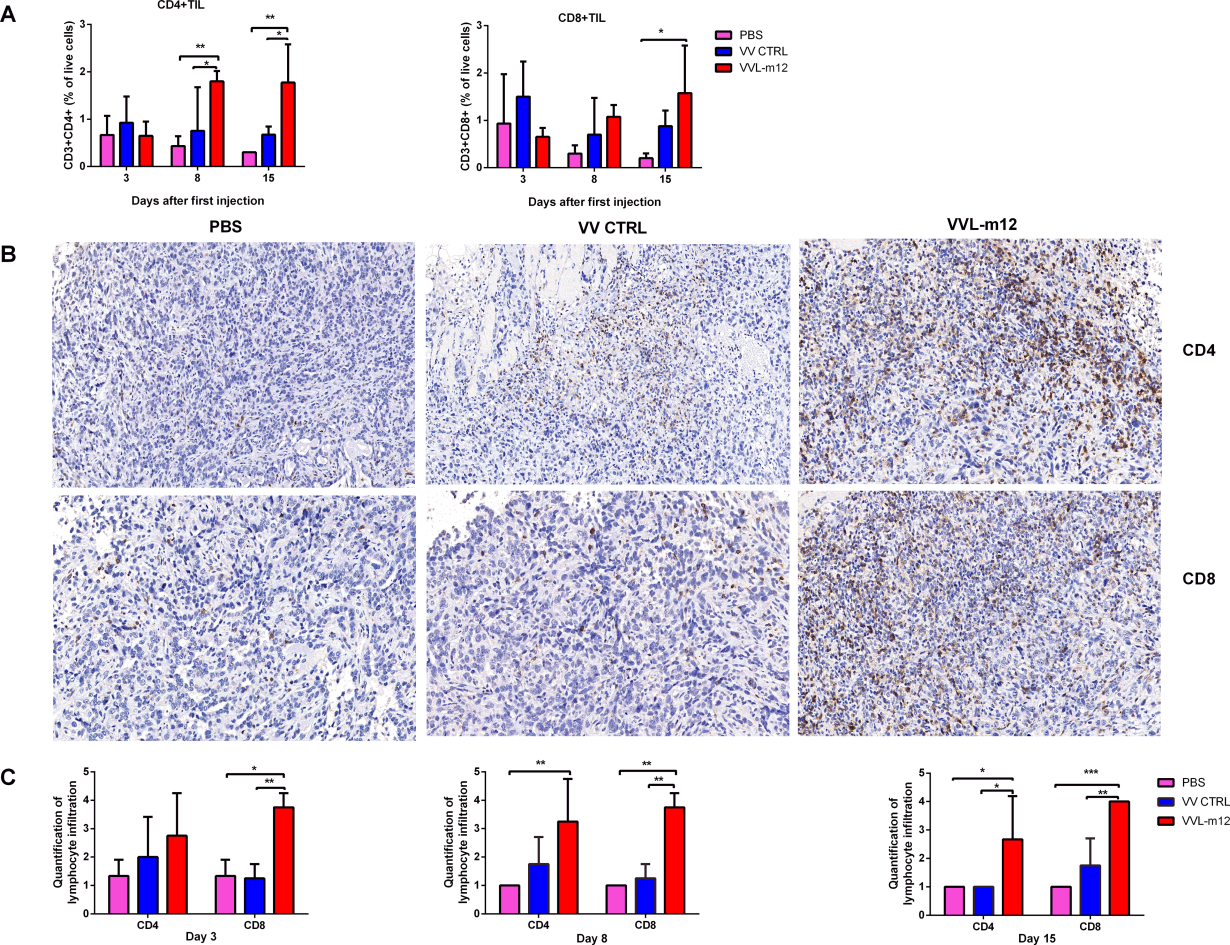
VVL-m12 therapy induces infiltration of CD4+ T and CD8+ T cells into the CMT64 tumor. Subcutaneous CMT64 mice were treated with one I.T. injection (1×108 PFU) of VV CTRL, VVL-m12 or PBS on day 1. Tumors were harvested 3, 8 and 15 days after injection and analyzed using flow cytometry (FC) and immunohistochemical staining (IHC) (n= 3/group). (A) CD4+ and CD8+ populations as a percentage of live cells in tumor tissue of treated mice assessed by gating on CD3+CD4+ and CD3+CD8+, respectively. (B) Representative images of IHC staining for CD4+ T and CD8+ T cells in CMT64 subcutaneous tumors collected on day 8 (×200), n=3/time point. (C) CD4+ T and CD8+ T cells were counted in five high-power fields (HPFs) from each tumor section (×200). Quantitative scores of lymphocyte infiltration within the tumor were assessed on days 3, 8 and 15 after therapy. The extent of positive cells was categorized into the following four grades: 1, <15 cells/HPF; 2, 16-30 cells/HPF; 3, 31-45 cells/HPF; 4, > 45 cells/HPF. Significance was determined by two-way ANOVA with Tukey’s multiple comparison test. *p<0.05; **p<.01; ***p<0.001. PBS, phosphate-buffered saline.

The original version of this article has been updated.

