# Supplementary material for: A novel oncolytic Vaccinia virus armed with IL-12 augments antitumor immune responses leading to durable regression in murine models of lung cancer
Source: Front Immunol. 2025 Jan 7;15:1492464. doi: 10.3389/fimmu.2024.1492464 (PMC11747717; doi:10.3389/fimmu.2024.1492464)
Supplement: Supplementary file 1 [file DataSheet1.docx]

**A novel oncolytic Vaccinia virus armed with IL-12** **augments antitumor immune responses leading to durable regression in murine models of lung cancer**

**Supplementary Materials and Methods**

*Real-time quantitative PCR*

Subcutaneous tumors as well as organs including lung, kidney and liver collected from treated animal were homogenized. DNA extraction was performed using QIAamp DNA Blood Mini Kit (Qiagen Ltd.) according to the manufacturer's instructions. Quantification of viral genome copy number was achieved using the TaqMan® PCR system provided by Applied Biosystems. For VV quantification, the primers and probe were designed for the vaccinia virus later transcription factor 2 (VLTF-2) gene: Probe: 6-FAM-ATTTTAGAACAGAAATACCC-MGB; Primers: S 5’-AACCATAGAAGCCAACGAATCC-3’, AS 5’-TGAGACATACAAGGGTGGTGAAGT-3’. Samples, controls, and standards were tested in triplicate by quantitative polymerase chain reaction (qPCR) using 7500 Real-time PCR System. Results were normalized to NanoDrop readings and expressed as genome copy number/0.01 g tissue. One-way ANOVA followed by Bonferroni post-test was used to assess significance.

**Investigation of macrophage polarization using bone marrow derived macrophages**

Femur and tibia bones were harvested from 6- to 8-week old mice. Bone marrow was flushed out through a 70μm cell strainer with IMDM supplemented with 10% Fetal Calf Serum (FCS), then mixed with ACK (100μl/bone) to lyse red blood cells. Flushed cells were resuspended in 20ml IMDM, 10% FCS, 1% Penicillin/Streptavidin, 1% Glutamine and left to adhere overnight in 5% CO2 at 37℃. After cell incubation, supernatant was removed, non-adherent cells were spun down. Cells were resuspended at the concentration of 0.5×10^6^ cells/mL in complete bone marrow macrophage medium (IMDM, 20 ng/mL M-CSF, 10% FCS) using a suspension culture dish (Corning, 10mL/dish) and cultured for 7 days. Then cells were lifted and re-plated for stimulations and analysis. Cell were plated in 24 wells plate at a final concentration of 2.5×10^5^ cells/mL and stimulated with IFN-γ (20 ng/mL) and LPS (100 ng/mL) or IL-4 (20 ng/mL) for 24 hours. Then cells were washed with PBS and infected with virus using an MOI of 1 PFU/cell for 24 hours. For cancer cell addition, CMT64 and DT6606 cells, at a ratio of 1 macrophage: 2 cancer cells were added to macrophages 4 hours before virus infection. 24 hours later, cells were collected for macrophage polarization analysis using FACS. Samples were seeded in triplicate and two biological repeats carried out. Macrophages are defined as CD11b+F4/80+ cells. M1 macrophages are MHCII+CD206-CD11b+F4/80+ cells, whereas M2 macrophages are CD206+CD11b+F4/80+ cells. Percentage of innate cells is determined by one-way ANOVA with Bonferroni post-test.





**Supplementary figure 1.** **A novel tumor-selective oncolytic VVL-h12 replicates in and kills human lung cancer cells.** (**A)** Cytotoxicity of VVLΔTKΔN1L, VV CTRL and VVL-h12 against human lung cancer A549, H460 and H1299 cell lines. Cell death was determined by MTS assay 144 hours post-infection. Mean EC50 values ± SEM are shown. One-way ANOVA with post hoc Tukey tests were used to assess significance. **(B-C)** Tumor selectivity was confirmed by assessing replication of VV CTRL and VVL-h12 in normal human bronchial epithelial cells (NHBE). **(D-E)** Virus replication was determined in human lung cancer cell lines including A549, H460 and H1299 cells. Virus production from whole cell lysates was assessed over 72 hours **(D)** and EEV production was determined via titration of the viral supernatant from the same experiments over 48 hours **(E)**. Mean PFU/cell ± SEM was shown at each time-point and statistical significance determined using two-way ANOVA with Tukey’s multiple comparison post-test. **(F)** hIL-12 expression after infection of human lung cancer cell lines at an MOI of 1 PFU/cell. Supernatant was collected every 24 hours for 72 hours and assayed for IL-12 by ELISA. Data were normalized to cell number infected and displayed as ng/2×10^5^ cells. In all cases, the mean ± SEM is shown. *p<0.05; **p<0.01; ***p<0.001; ****p<0.0001.


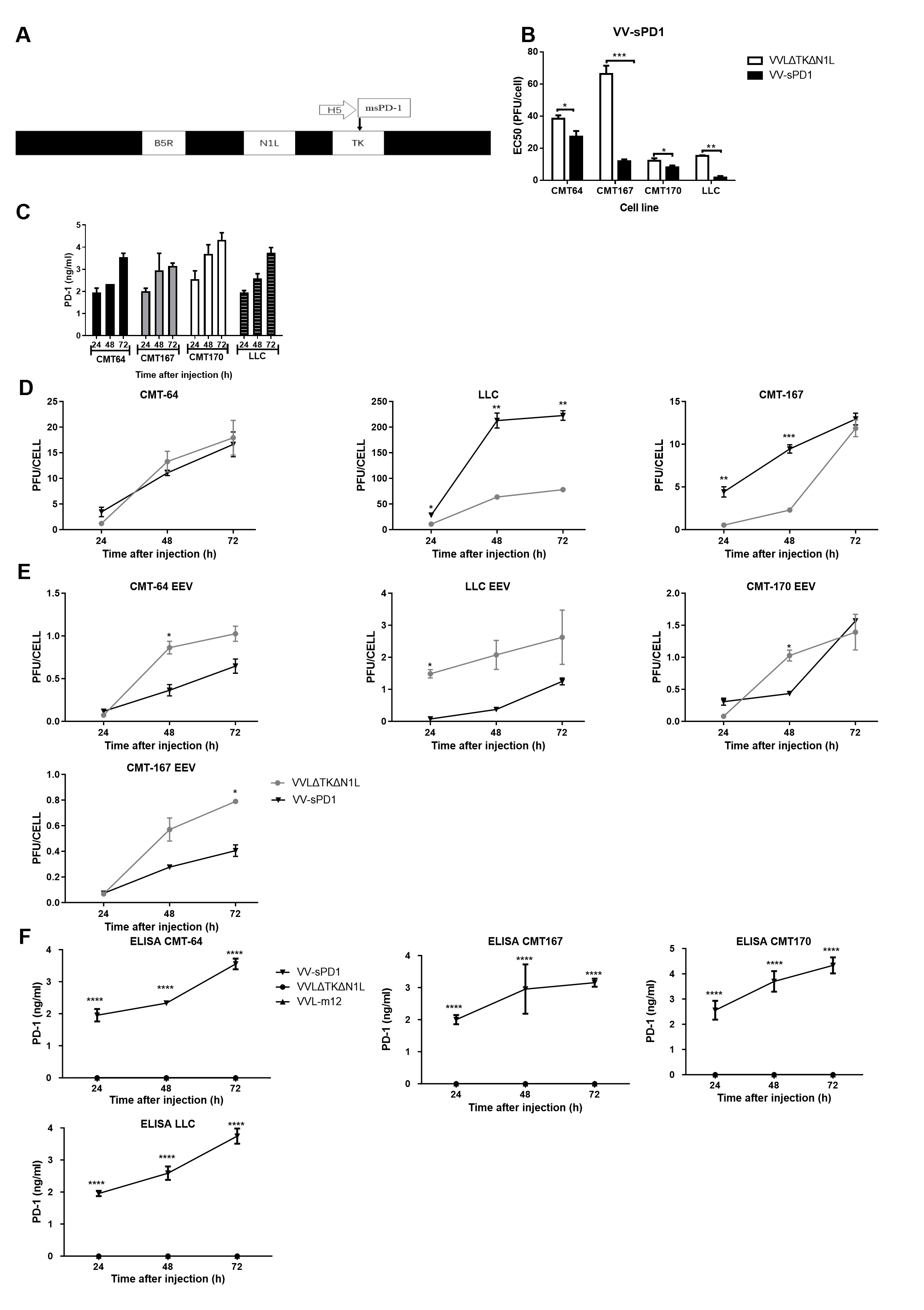


**Supplementary figure 2.** **Insertion of the mouse soluble PD1 into the TK region of VV can replicate in and kill mouse lung cancer cell lines.** (**A)** Vaccinia virus Lister strain with deletions of the thymidine kinase (TK) and N1L genes, has been described previously. This virus was further modified by insertion of mouse soluble PD1 (msPD1) into TK region under control of the endogenous H5 promoter as shown. **(B)** The EC50 value (dose required to kill 50% of cells) of VVLΔTKΔN1L and VVΔTK-ΔN1L-msPD-1 (referred to hereafter as VV-msPD1) was compared using MTS assays in murine (CMT64, CMT167, CMT170 and LLC) lung cancer cell lines. Mean EC50 values ± SEM are shown and a student unpaired T test used to assess significance (n=3/group). **(C, F)** PD-1 expression after infection of murine lung cancer cell lines at an MOI of 1 PFU/cell. Supernatant was collected every 24 hours for 72 hours and assayed for mouse PD-1 by ELISA. Data were normalized and displayed as ng/ml (n=3/group). **(D-E)** Virus replication was determined in murine lung cancer cell lines including CMT64, CMT167, CMT170 and LLC cells. Virus production from whole cell lysates was assessed over 72 hours **(D)** and EEV production was determined via titration of the viral supernatant from the same experiments **(E)**. Mean PFU/cell ± SEM was shown at each time-point and statistical significance determined using a student unpaired T test at each timepoint. In all cases, the mean ± SEM is shown (n=3/group). *p<0.05; **p<0.01; ***p<0.001; ****p<0.0001.


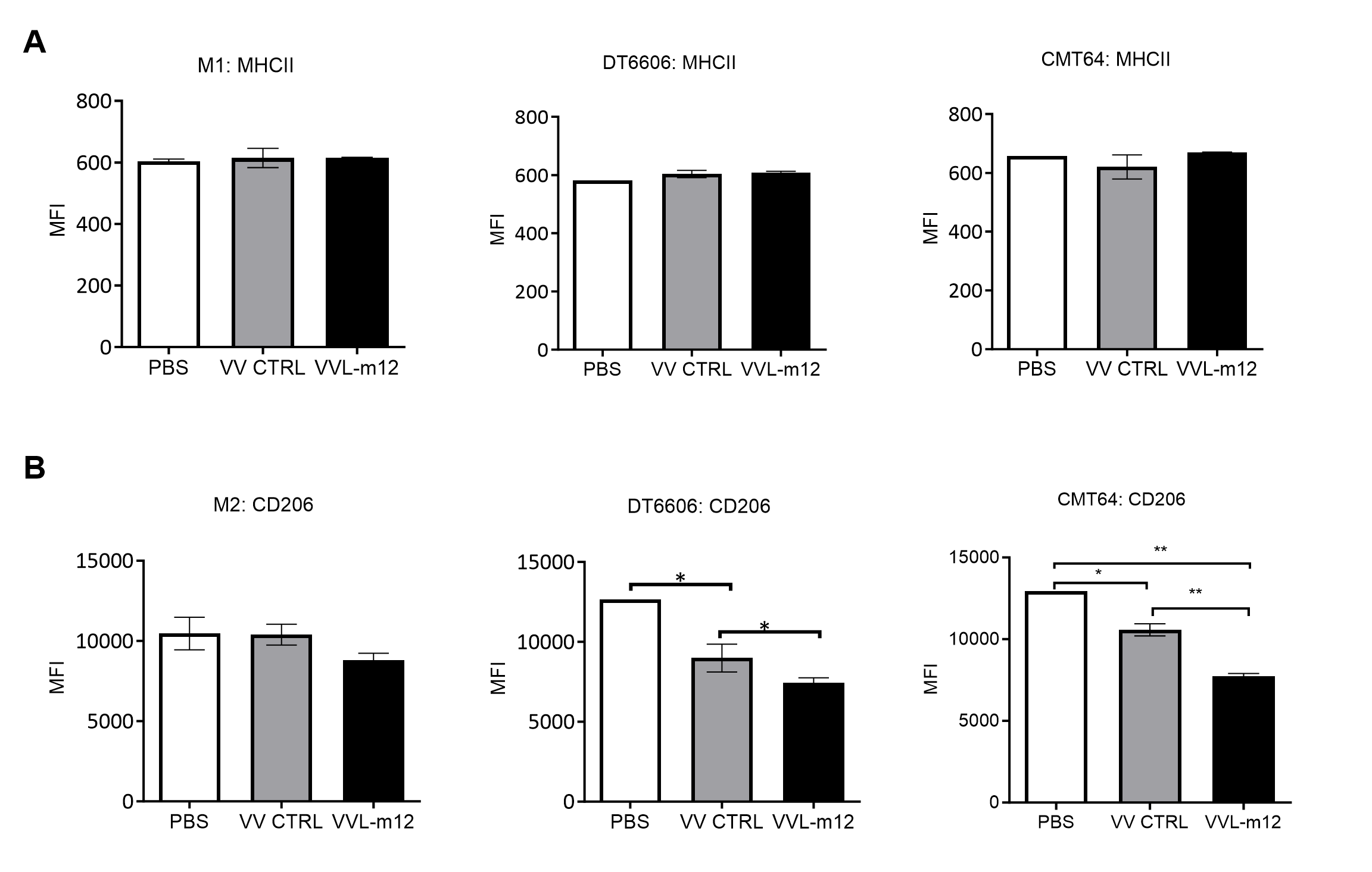


**Supplementary figure 3. VVL-m12 treatment significantly reduces M2 macrophages in vitro. (A-B)** Schematic detailing in vitro isolation and culture of bone marrow-derived macrophages. MHCII^high^ macrophages were considered M1 polarized and CD206^high^ considered M2 polarized. Median fluorescence intensity (MFI)±SEM is shown and results analyzed using a one-way analysis of variance (ANOVA) with Bonferroni post-test.


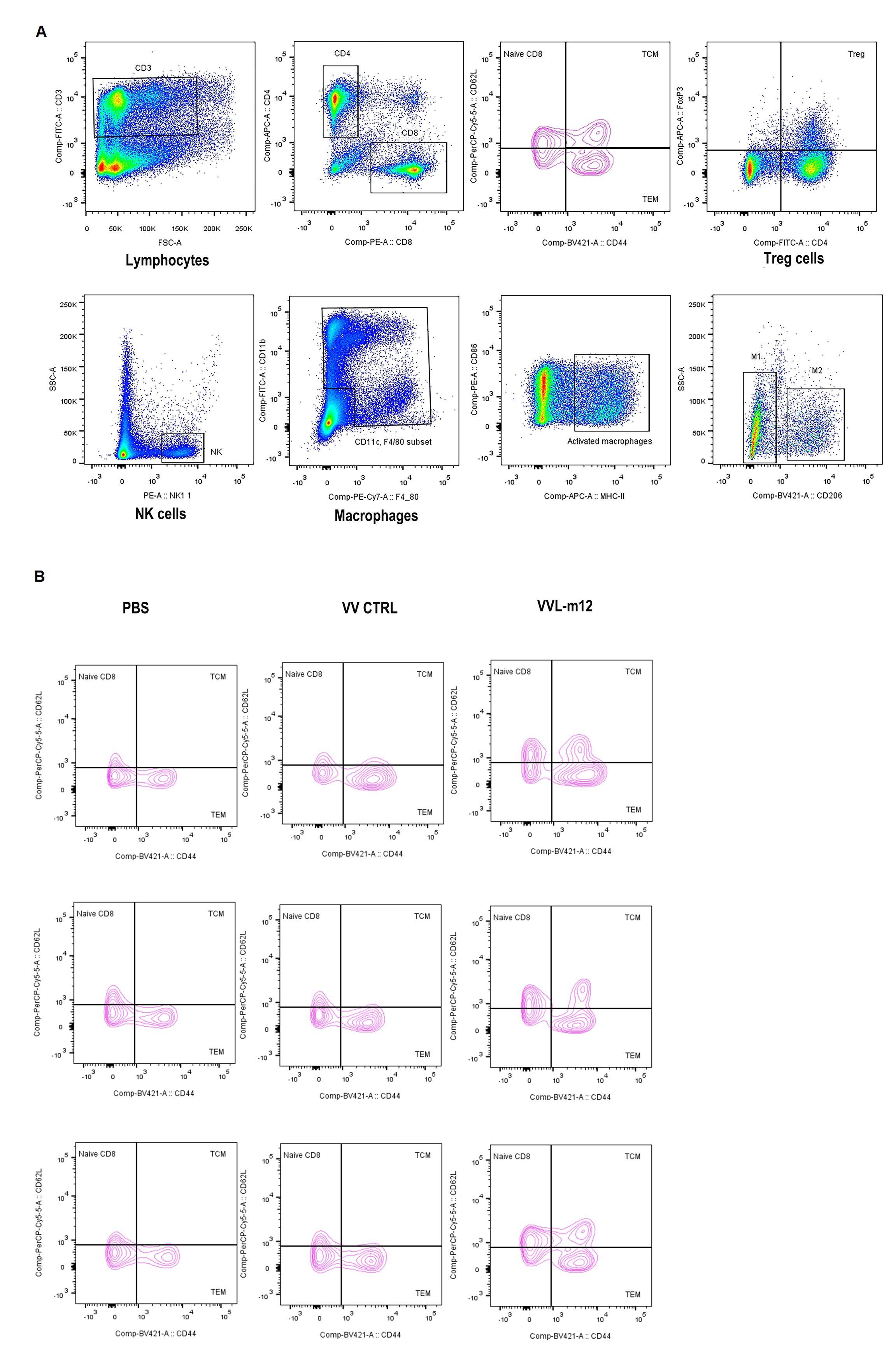


**Supplementary figure 4. VVL-m12 induces adaptive and innate immunity against lung cancer in vivo.** CMT64 tumors were established as previously and treated once with 1×10^8^ PFU VV CTRL or VVL-m12 or PBS for control. Spleens were harvested 3, 8 and 15 days after treatment. **(A)** The flow cytometry gating strategy for adaptive and innate immune populations is shown. **(B)** Representative flow cytometry profiles with CD8+ TCM populations as a percentage of CD8+ T in splenocytes of treated mice.


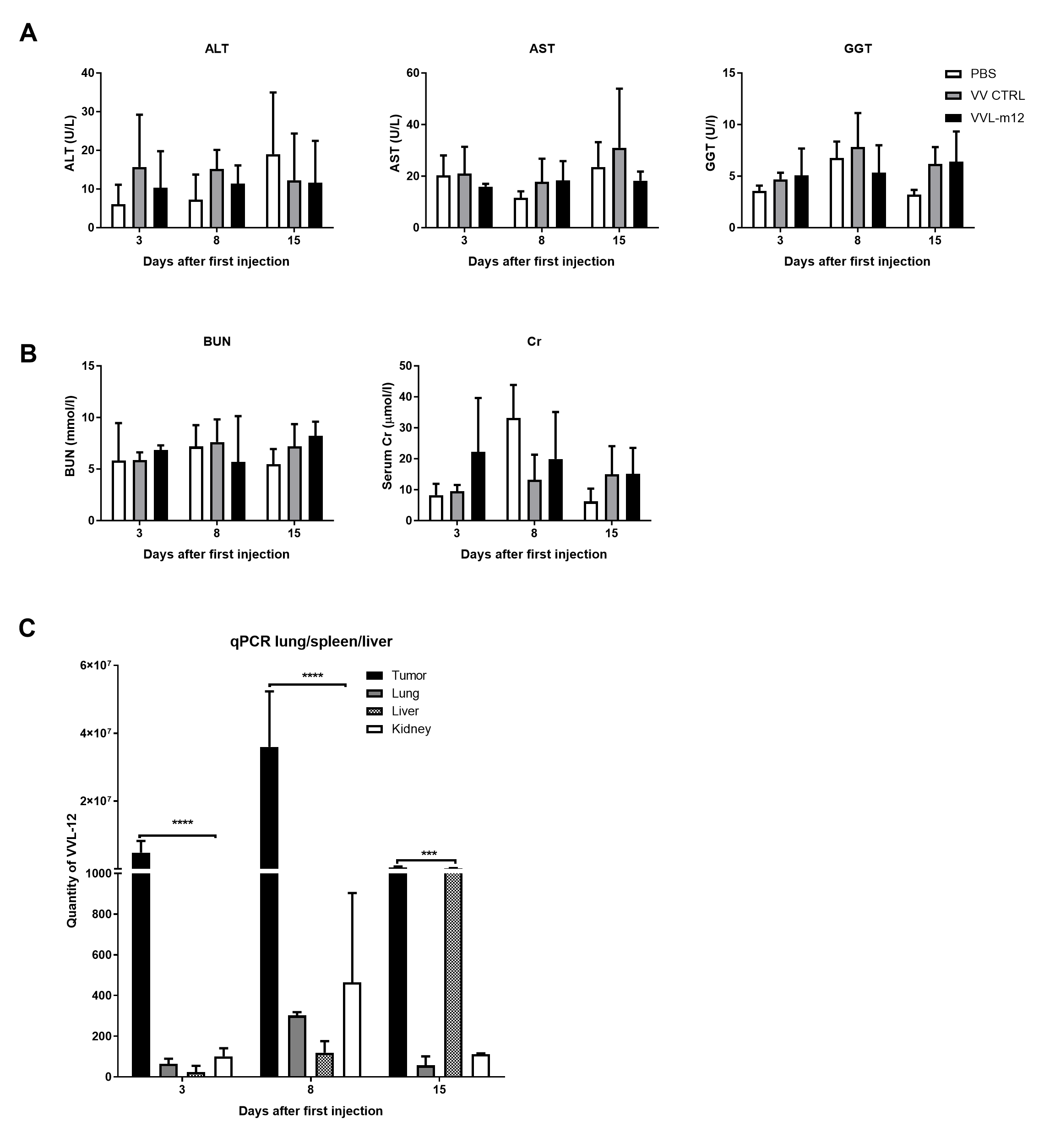


**Supplementary figure 5. VVL-m12 is a safe treatment for lung cancer with high selectivity for cancer.** CMT64 tumors were established as previously and treated once with 1×10^8^ PFU VV CTRL or VVL-m12 or PBS for control. **(A-B)** On day 3, 8 and 15, Blood samples are collected from the ocular venous plexus of each mouse for assessing the influence of VVs on hepatic and renal function consist of serum alanine aminotransferase (ALT), aspartate aminotransferase (AST), gamma glutamyl transpeptidase (GGT), creatinine (Cr), blood urea nitrogen (BUN) using ELISA*.* **(C)** Subcutaneous tumors, lungs, livers, and kidneys were harvested from three mice in each group for quantitative PCR to detect vaccinia virus later transcription factor 2 (VLTF-2) gene. In all cases, the mean ± SEM is shown at each time-point and statistical significance determined using two-way ANOVA with Tukey’s multiple comparison post-test. *p<0.05; **p<0.01; ***p<0.001; ****p<0.0001.
